# In vitro fertilization and a patient in compulsory psychiatric treatment in the community

**DOI:** 10.1192/j.eurpsy.2022.921

**Published:** 2022-09-01

**Authors:** A. Pirtovsek Savs, M. Derganc

**Affiliations:** Psychiatric Clinic ljubljana, Ckp, ljubljana, Slovenia

**Keywords:** Abortion, COMPULSORY PSYCHIATRIC TREATMENT IN THE COMMUNITY, IVF, schizophrénia

## Abstract

**Introduction:**

INTRODUCTION: According to the Universal Declaration of Human Rights, everyone has the right to start a family. Under the Slovenian Infertility Treatment Act, everyone has the right to infertility treatment. A case of a patient in compulsory psychiatric treatment who participated in the process of IVF is presented.

**Objectives:**

CASE REPORT: A 40-year-old male with paranoid schizophrenia has already been hospitalized thirteen times. He often discontinued therapy, abused drugs and repeatedly exhibited violent behaviour. He already had a child from a past relationship he didn’t care of.

**Methods:**

During the compulsory psychiatric treatment ordered by the court his mental status improved because his treatment with antipsychotics was supervised. He was in a relationship with a thirty-year-old partner. After unsuccessful attempts to become pregnant, they expressed a desire to conceive with biomedical assistance. Their application was considered and approved by the IVF Commission.

**Results:**

The procedure was successful but in the 13th week of pregnancy, the patient’s partner changed her mind due to his aggression. Because she was pregnant for more than 10 weeks, she had to submit a request for artificial termination to the Commission for abortion. Her request was granted and the pregnancy was terminated.

**Conclusions:**

CONCLUSION: We live in time of endless possibilities. Despite of violent acts in the past and severe form of mental illness, the couple was granted IVF procedure. Everyone has the right to start a family; however, the question that has to be raised is the extent and reasonableness of involvement of medical profession and/or health care system.

**Disclosure:**

No significant relationships.

